# Housing modification for malaria control: impact of a “lethal house lure” intervention on malaria infection prevalence in a cluster randomised control trial in Côte d’Ivoire

**DOI:** 10.1186/s12916-023-02871-1

**Published:** 2023-05-04

**Authors:** Jackie Cook, Eleanore Sternberg, Carine J. Aoura, Raphael N’Guessan, Immo Kleinschmidt, Alphonsine A. Koffi, Matthew B. Thomas, Serge-Brice Assi

**Affiliations:** 1 International Statistics and Epidemiology Group, London School of Hygiene and Tropical Medicine, London, UK; 2 Tropical Health LLP, London, UK; 3 Institut Pierre Richet, Bouaké, Côte d’Ivoire; 4 Department of Disease Control, London School of Hygiene and Tropical Medicine, London, UK; 5 Wits Research Institute for Malaria, School of Pathology, University of Witwatersrand, Johannesburg, South Africa; 6 Southern African Development Community Malaria Elimination Eight Secretariat, Windhoek, Namibia; 7 Department of Entomology & Nematology and the Invasion Science Research Institute, University of Florida, Gainesville, USA; 8 Institut National de Sante Publique, Abidjan, Côte d’Ivoire

**Keywords:** Malaria, Housing, Vector control, Lethal house lure, Intervention, Trial, Diagnostics

## Abstract

**Background:**

In recent years, the downward trajectory of malaria transmission has slowed and, in some places, reversed. New tools are needed to further reduce malaria transmission. One approach that has received recent attention is a novel house-based intervention comprising window screening (S) and general house repairs to make the house more mosquito proof, together with EaveTubes (ET) that provide an innovative way of targeting mosquitoes with insecticides as they search for human hosts at night. The combined approach of Screening + EaveTubes (SET) essentially turns the house into a ‘lure and kill’ device.

**Methods:**

This study evaluated the impact of SET on malaria infection prevalence in Côte d’Ivoire and compares the result in the primary outcome, malaria case incidence. Malaria infection prevalence was measured in a cross-sectional survey in 40 villages, as part of a cluster-randomised trial evaluating the impact of SET on malaria case incidence.

**Results:**

Infection prevalence, measured by rapid diagnostic test (RDT), was 50.4% and 36.7% in the control arm and intervention arm, respectively, corresponding to an odds ratio of 0.57 (0.45–0.71), *p* < 0.0001). There was moderate agreement between RDT and microscopy results, with a reduction in odds of infection of 36% recorded when infection was measured by microscopy. Prevalence measured by RDT correlated strongly with incidence at a cluster level.

**Conclusions:**

In addition to reducing malaria case incidence, house screening and EaveTubes substantially reduced malaria infection prevalence 18 months after installation. Infection prevalence may be a good metric to use for evaluating malaria interventions in areas of similar transmission levels to this setting.

**Trial registration:**

ISRCTN18145556, registered 1 February 2017.

**Supplementary Information:**

The online version contains supplementary material available at 10.1186/s12916-023-02871-1.

## Background

Vector control has been credited with a large contribution to the decline in malaria cases and deaths in the past 15 years [1]. However, the effectiveness of the two main interventions targeting anopheline vectors, insecticide-treated nets (ITN) and indoor residual spraying (IRS), is threatened by progressively increasing resistance to pyrethroid insecticides [2, 3]. This threat has necessitated the development of new vector control tools using novel delivery methods or alternative insecticides [4].

One such tool is the In2Care® EaveTube. EaveTubes comprise PVC tubes which are inserted into a house at eave level and are closed off using an insecticide treated netted insert. EaveTubes exploit the preference of the anopheline mosquito to enter households at eave height [5] and have been termed by WHO as a ‘lethal house lure’ [6] due to their ability to both attract (by funnelling human odours) and kill (through contact with insecticide) mosquitoes [7, 8].

Initial entomological studies conducted in semi-field enclosures suggested that the combination of EaveTubes, closed eaves, and screened windows successfully reduced mosquito populations [9, 10]. To establish whether this would translate into reduced malaria case incidence, a cluster randomised control trial was undertaken between 2017 and 2019 in central Côte d’Ivoire [11]. The trial generated convincing evidence that the use of house screening and EaveTubes (a combination referred to as SET) reduced malaria transmission, with close to a 40% drop in malaria case incidence in children aged 6 months to 10 years living in intervention clusters, compared to control clusters [11].

The incidence of clinical malaria (new symptomatic malaria cases recorded in a population) is typically considered the gold standard measure of impact for malaria intervention trials [12], and it can be measured either through passive or active case detection. A rigorous passive case detection system uses data collected from attendees at health facilities; however, this kind of data collection can be difficult to implement and relies on a robust existing health information system. Active case detection is likely a more accurate method of measuring incidence, but it requires regular visits to a defined cohort of participants, which is logistically complicated and expensive to undertake.

Measuring infection prevalence incurs vastly fewer cost implications for this type of evaluation compared to the measurement of incidence. Prevalence can be estimated through cross-sectional surveys at single time points which greatly reduces time, expense, and logistical burden. As part of the evaluation of the SET technology, we performed an endline cross-sectional survey to assess the impact on malaria infection prevalence in the study population 18 months post-installation of SET. This paper evaluates the impact of a vector control intervention on malaria infection prevalence using different diagnostics in a trial where SET resulted in a substantial reduction in malaria case incidence in the intervention clusters.

## Methods

### Study site

The trial took place in 40 villages (clusters) within 50 km of the city of Bouaké, central Côte d’Ivoire. The study setting has moderate to high malaria transmission with seasonal transmission primarily occurring between May to November each year. There is a high level of pyrethroid resistance in the local anopheline vectors, as well as resistance to carbamates and organochlorides [13].

### Trial overview

The protocol and primary results for the trial have been previously published [11, 14]. Briefly, the trial took place in 40 clusters (villages with between 100 and 600 houses), covering a population of approximately 50,000 people. A baseline survey was done in August 2016 prior to the start of the trial in which approximately 60 children aged 6 months to 10 years from each cluster were randomly selected for testing for malaria using rapid diagnostic tests (RDT), regardless of symptoms. A short questionnaire was administered to the head of household to collect information on house type and structure, socioeconomic status, intervention use, and education levels. Restricted randomisation was used to ensure balanced allocation of clusters to study arms with respect to cluster size, proportion of households suitable for the intervention, and presence of a health facility within a cluster, as well as to household socioeconomic status, malaria infection prevalence in cohort aged children, and insecticide-treated net use. Clusters were allocated 1:1 to the control arm (ITN only) or the intervention arm (ITN, house screening and EaveTube installation (SET)). Households in the intervention clusters had the option to have SET installed if their house was of a suitable material to withstand the installation process (brick walls, tin roof). The intervention was installed between October 2016 and February 2017, at which point mass distribution of pyrethroid-only nets took place in all study villages. Coverage of SET in intervention clusters ranged from 32 to 100%, with a mean coverage of 72% of houses (Additional file 1).

The main epidemiological outcome of the trial was malaria case incidence. This was measured in a cohort of 50 children aged 6 months to 10 years per cluster. The cohorts were visited every 2 weeks during the transmission season (May to November) and once a month during the dry season (December to April). Febrile children were tested for malaria with an RDT and received treatment (artesunate-amodiaquine) if positive. The full analyses of the incidence data, as well as entomological and economic outcomes, have been published elsewhere [11].

### Endline prevalence survey

An endline cross-sectional survey took place in November 2018, 18 months after the installation of SET. In contrast to the baseline survey where households were the sampling unit, compounds (ranging from 1 to 5 households) were randomly selected from census lists of each cluster, and individuals were tested until approximately 70 people per cluster had been sampled. Anyone over the age of 6 months was invited to be part of the study. Participants were tested using RDT (SD Bioline Malaria Ag P.f/Pan; Standard Diagnostics; Seoul, South Korea) and had a blood smear taken for microscopy analysis, regardless of symptoms of malaria. A short questionnaire was also administered to collect information on house structure, household assets, intervention use, and potential malaria symptoms.

The prevalence survey had 80% power to detect a 40% reduction in prevalence between the control arm and the SET arm, assuming a 40% prevalence in the control arm and a coefficient of variation between clusters of 0.5.

### Statistical methods

Descriptive statistics (proportions and means) and infection status associations were calculated with confidence intervals adjusting standard errors for clustering at the village level using the svy command in Stata. To assess the impact of the intervention, a mixed effect logit model with arm as a fixed effect and cluster included as a random effect was used to produce odds ratios using both the RDT and/or microscopy data. Impact was assessed separately for villages with more than 70% of houses with SET installed, compared to those with less than or equal to 70% coverage. Per protocol analyses involved comparison of children who lived in houses with SET installed with those in control clusters.

To compare differences between the baseline and endline results (in children aged 6 months to 10 years only), an interaction between arm and survey was fitted to the mixed effect logistic model previously described.

Incidence was calculated as described in detail in the main trial results paper [11]. Cluster-level endline prevalence was compared with cluster-level incidence after 2 years of follow-up using linear regression to assess the association between these two measures.

This study is registered as an International Standard Randomised Controlled Trial, ISRCTN18145556.

## Results

### Baseline survey results

In the baseline survey, 2559 children aged 6 months to 10 years from 1217 households were tested for malaria. The mean age of children tested was 5 years. Similar proportions of males and females were tested. The majority of children reported always using a bed net (76.8%), whilst 19% reporting never using a net. Children aged 5–10 years were more likely to report never using a net (22.6%), compared to 0–2 years (15.7%) or 2–5 years (16.5%). 73.2% (*n* = 1851) of children were infected by malaria (determined using RDT) at the time of the survey. Children aged 5–10 years were more likely to be RDT positive (76.9%), compared to 0–2 year olds (63.8%) and 2–5 year olds (72.7%). The results from the baseline survey were used for the restricted randomisation, to ensure that the two arms were balanced on infection prevalence (Table 1). In August 2016, the baseline mean cluster infection prevalence for the study area was 72.4 (range: 45.3–95.5) in children aged 6 months to 10 years.

**Table 1:** Population summary of participants in the baseline and endline survey by arm

	**Baseline survey (July 2016)**		**Endline survey (Nov 2018)**	
	Control	SET	Control	SET
**Clusters**	20	20	20	20
**Households**	625	592	134	141
**SES (households) (*n*, %)**				
** Lowest**	246 (41.8)	162 (30.0)	66 (49.3)	43 (30.5)
** Middle**	177 (30.1)	173 (31.9)	33 (24.6)	46 (32.6)
** Highest**	165 (28.1)	207 (38.2)	35 (26.2)	52 (36.9)
**Households with SET**	-	-	-	106 (75.2)
**Individuals**	1268	1290	1414	1429
**Median age (years)**	5	5	12	13
**Age group (years); *n* (%)**				
** 0–5**	706 (55.6)	703 (54.4)	331 (23.4)	317 (22.2)
** 5–10**	562 (44.3)	587 (45.5)	283 (20.0)	307 (21.5)
** 10–15**			188 (13.3)	146 (10.2)
** 15–25**			124 (8.8)	135 (9.5)
** 25–50**			268 (19.0)	291 (20.4)
** 50–100**			220 (15.6)	233 (16.3)
**Sex**				
** Female; *n* (%)**	629 (49.8)	677 (52.6)	827 (58.5)	834 (58.4)
**People sleeping under a net the night before; *n* (%) **	978 (77.8)	970 (75.8)	1090 (77.1)	971 (68.0)
**Baseline prevalence (August 2016) in children aged 6 months to 10 years, RDT; % (95% confidence intervals)**	73.6 (45.3–91.1)	71.3 (48.4–95.5)		

### Endline survey results (18 months post-installation)

For the endline survey, 2843 people from 275 compounds were included across the 40 clusters. The median age of participants was 12 years old (range: 6 months to 98 years). Net use was relatively high, with 72.5% of people reporting using a net the previous night, though this appeared lower in the intervention arm (68.0%) compared to the control arm (77.1%). The difference in net use across age groups was greater in the SET arm (ranging from 55% in 10–15 year olds to 76% in 50–100 year olds) compared to the control arm (ranging from 71% in 10–15 year olds to 84% in 0–5 year olds).

In the endline survey, mean prevalence by cluster was 43.5% (95% CI: 23.7–66.2). Infection was highest in males (47.4% vs 40.8% in females) with the burden of infection highest in children aged 5–10 years (66.8%). Infection was highest in individuals living in households classified as the lowest SES category (46.9%, compared to 43.5% and 40.1% in the middle and higher categories). Infection was lower in those who reported using a net the previous night (41.0% compared to 50.0%).

### Impact of Screening and EaveTubes (SET) on infection prevalence measured by RDT

**Fig. 1:**
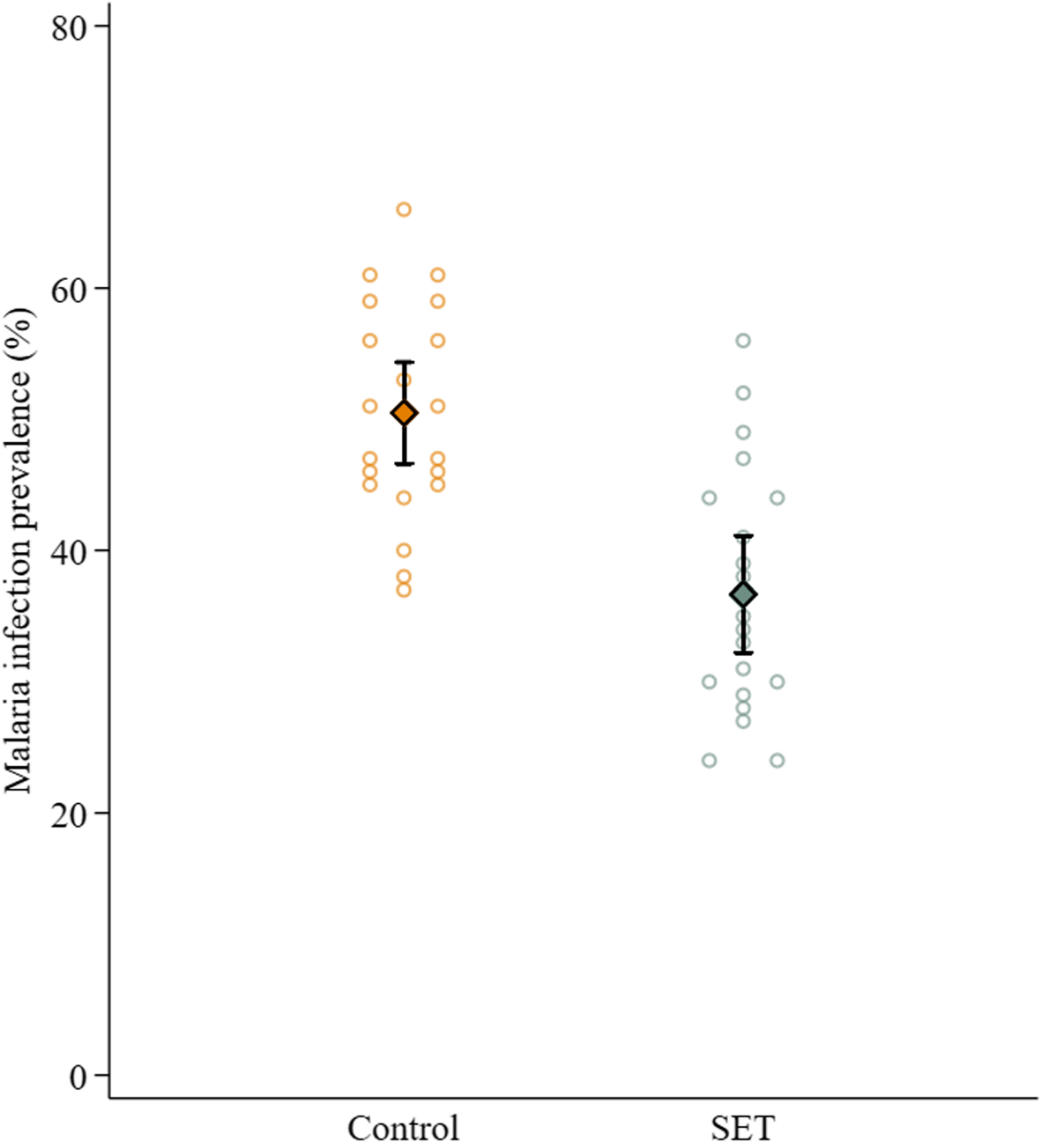
Infection prevalence in each cluster (open circles) by intervention arm. The mean of the cluster results and 95% confidence intervals are shown in the diamonds

Infection prevalence was lower in the intervention arm (36.7%) compared to the control arm (50.4%), with 43% lower odds of infection in the SET arm compared to the control arm (odds ratio (OR) 0.57 (0.45–0.71), *p* < 0.001) (Table 2; Fig. 1).

**Table 2:** Impact of intervention on infection prevalence (measured using RDT)

	**Baseline infection prevalence in children aged 6 months to 10 years, % (95% CIs)**	**Endline infection prevalence in children aged 6 months to 10 years, % (95% CIs)**	**Prevalence difference (endline to baseline) (difference, 95% CIs), *p* value)**	**Endline prevalence in all ages, % (95% CIs)**	**Odds ratio (endline only), 95% CIs, *p* value **
Control	73.9 (67.7–79.3)	69.4 (63.5–74.6)	4.6 (− 3.3–12.4), *p* = 0.254	50.4 (46.8–54.0)	Reference
SET	72.4 (66.1–77.9)	51.0 (45.8–56.1)	21.4 (13.8–29.0), *p* < 0.001	36.7 (32.5–40.8)	0.57 (0.45–0.71), *p* < 0.001

In the intervention clusters, there was a reduction in malaria infection prevalence for all age groups compared to those living in control clusters. Individuals reporting sleeping under a net the night before had lower infection prevalence in both arms. There was no evidence for an interaction between intervention arm and bed net use, suggesting that the benefit of living in a SET village was additional (not multiplicative) to the protection conferred by a bed net. However, individuals living in SET villages but not using a bed net had lower prevalence (42.8%) than those using a net in control villages (47.5%) with the biggest difference between those living in control villages and not using a net (60.2%) and those living in SET villages and using a net (33.8%).

Due to the range of coverage of SET in the intervention clusters (32% to 100% of households), we assessed the impact of intervention coverage on malaria infection prevalence. Seven clusters had SET coverage of 70% or lower. At baseline, infection prevalence in the 7 lower coverage clusters was similar to infection prevalence in the 14 clusters which had > 70% coverage (infection prevalence of 72.3% (95% CI: 53.7–95.5) in clusters with coverage ≤ 70% and infection prevalence of 70.7% (95% CI: 48.4–88.5) in clusters with > 70% coverage). In the endline survey, clusters with ≤ 70% coverage had a mean infection prevalence of 43.4% (95% CI: 32.9–52.1) compared to a prevalence of 33.0% (95% CI: 23.7–56.3) in the clusters with coverage > 70%, suggesting a stronger effect of the intervention when coverage was higher. However, when comparing the lower coverage clusters to the control clusters, there was still evidence for a drop in prevalence in the lower coverage villages (OR 0.76 (95% CI: 0.60–0.95), *p* = 0.019). Importantly, the trial was not designed to assess the differential impact of coverage so these results should be interpreted with caution. A sensitivity analysis was performed controlling for cluster baseline prevalence and showed a similar result (OR 0.76 (95% CI: 0.59–0.98), *p* = 0.031).

Thirty-five (25%) of the households (297 individuals) sampled in the intervention arm did not have SET. Individuals living in these houses had comparable infection prevalence (37.4%) to those who did have the intervention (36.5%). When compared to individuals living in control villages, the impact of living in a SET cluster for those households without SET (OR 0.57 (0.40–0.83), *p* = 0.003) was similar to the impact for those households with SET. This suggests a community impact of the intervention, meaning that households benefited from others in their village having SET installed, regardless of whether they lived in houses with the intervention themselves.

### Infection prevalence at endline compared to baseline (RDT)

**Fig. 2:**
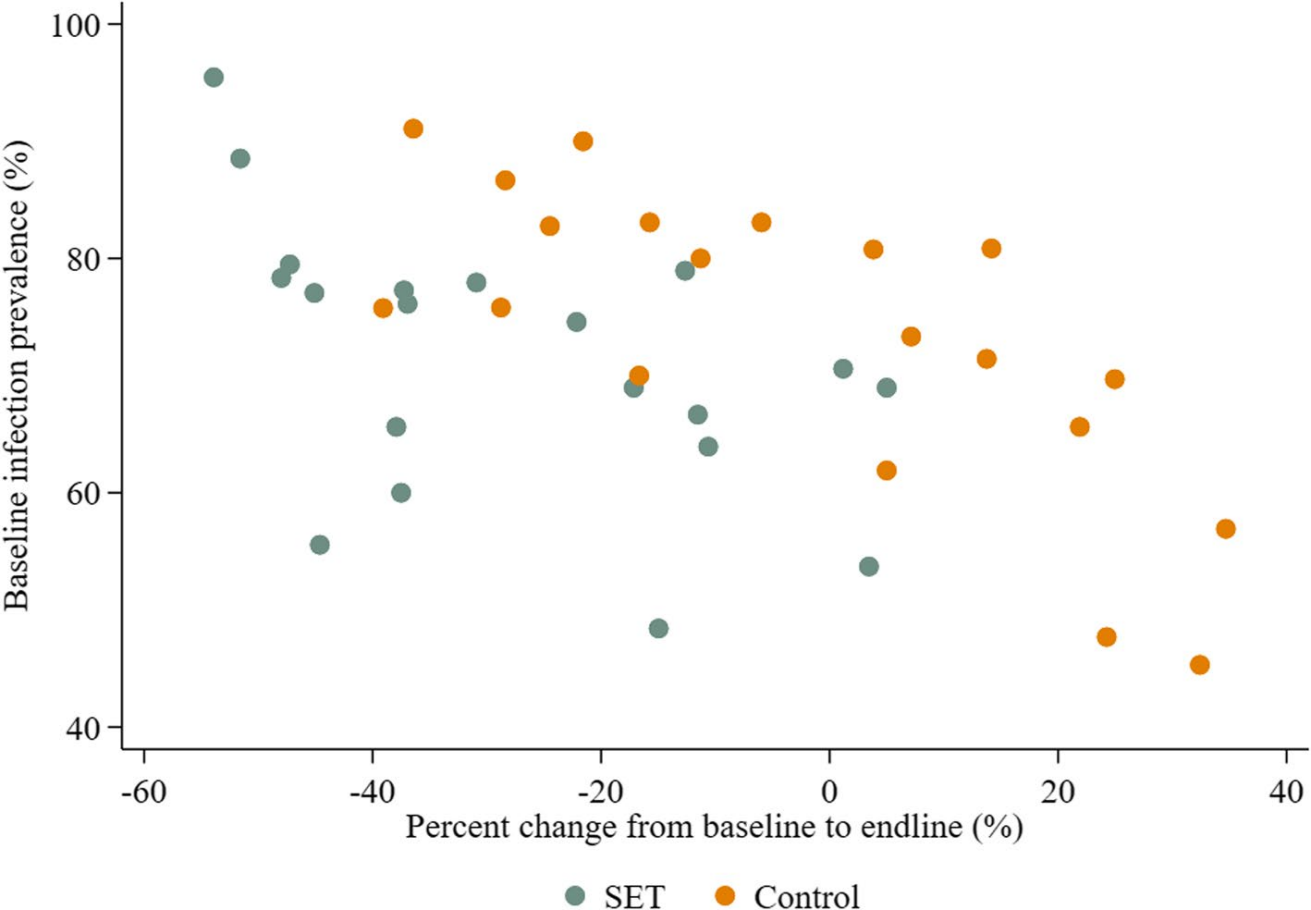
Percentage reduction in infection prevalence between endline and baseline surveys compared to baseline prevalence. Each circle represents a cluster

Infection prevalence in children aged 6 months to 10 years for each arm was compared between baseline and endline surveys to assess whether there was a reduction in either arm, with the caveats that the two surveys took place at different times of the year and had slightly different sampling methodologies. The interaction between study arm and survey was significant (*p* = 0.025). Prevalence in the control arm reduced from 73.9% (95% CI: 67.7–79.3) to 69.4% (95% CI: 63.5–74.6) (*p* = 0.254), whilst the prevalence in the intervention arm reduced from 72.4% (95% CI 66.1–77.9) to 51.0% (95% CI: 45.8–56.1), *p* < 0.001, corresponding to a risk difference of 21.4% (95% CI: 13.8–29.0) (Table 2). The reduction in prevalence was not universal across all intervention clusters, with 3 SET clusters having similar prevalences between baseline and endline (Fig. 2). SET coverage in these three villages was above 60%; however, net use the previous night was below 60% in all three. The largest percentage reductions between baseline and endline were seen in clusters where baseline prevalence was highest in both arms (Fig. 2).

### Comparison of diagnostics: RDT and microscopy

The majority of participants in the endline survey also had a blood smear taken for microscopy (*N* = 2725). 36.4% (*n* = 1072) of participants had a microscopically detectable infection, 98% of which were *Plasmodium falciparum* or mixed infections*. Plasmodium malariae* infections were detected in 2% of slides (*n* = 66), whilst *Plasmodium ovale* infections were detected in 0.4% of slides (*n* = 13). Similarly, to the RDT results, the prevalence of microscopic infections were lower in participants living in intervention clusters (31.9%) compared to those living in the control clusters (41.4%) (OR 0.64 (0.44–0.93), *p* = 0.019).

Overall, 57.3% (707/1233) of positive RDT results were also positive by microscopy. Whilst age patterns of infection prevalence appeared similar for the two diagnostics, the agreement between the two was lowest in older participants (only 36.5% and 31.1% of positive RDT results were positive by microscopy in 25–50 year olds and 50–100 year olds respectively). Mean parasite density was also lower in infections in those age groups (< 2000 trophozoites per ml) compared to younger age groups (mean 36,384, 12,300, 3789, 6437 trophozoites per ml in 0–5, 5–10, 10–15, and 15–25 year olds, respectively).

### Infection prevalence (RDT) compared to incidence

**Fig. 3:**
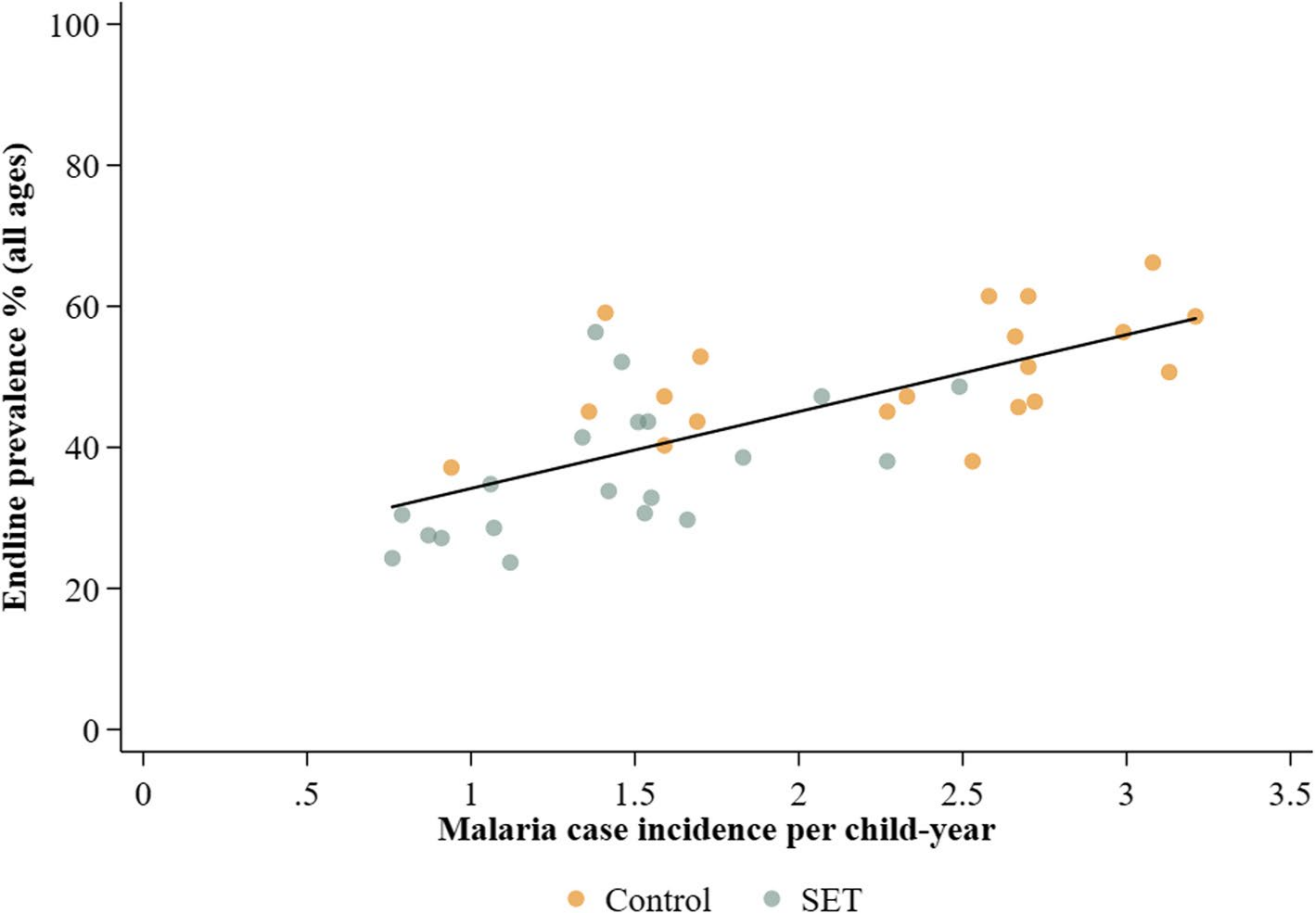
Association between endline prevalence (all ages) and malaria case incidence in children aged 6 months to 10 years

Cluster-level infection prevalence in the endline survey was associated with malaria case incidence measured in the child cohort at the end of the two years (Fig. 3). Cluster-level linear regression of case incidence on prevalence suggested that for every increase of 1 case per child per year, there was an increase of 10.9% in infection prevalence (measured by RDT) (7.3–14.5%), *p* < 0.001).

## Discussion

New paradigms of vector control tools are urgently needed if we are to continue the downward trajectory of malaria transmission that has been achieved over the past few decades. Housing modifications are a paradigm that has rarely been explored in cluster randomised trials. In this large trial, EaveTubes combined with house screening demonstrated a strong impact on malaria case incidence in Côte d’Ivoire [11]. The current analysis shows that a similarly strong impact was achieved on malaria infection prevalence measured 18 months after the introduction of the SET intervention, with a 43% and 36% reduction in odds of malaria infection across all age groups in prevalence when measured by RDT and microscopy, respectively. Additionally, an impact on prevalence was seen in residents living in houses without SET in the intervention arm, suggesting a community impact of the intervention.

The endline prevalence survey took place at the end of the transmission season, 18 months after the intervention had been installed. Bed net trials in Tanzania have shown that an intervention’s impact on prevalence can differ substantially in accordance with the timing of the survey, which may be a reflection of seasonality of transmission, or the ageing of the intervention [15]. In the control arm in this study, the endline data collected in November (end of transmission season) were similar to the baseline data, collected in August, despite the difference in timing and sampling. This suggests a reduction in malaria transmission occurred in the control arm as well, as we would hypothesise that prevalence would be higher in November than August if no interventions had been in place because prevalence tends to increase throughout a transmission season as more people become infected. This implies that the pyrethroid-treated bed nets distributed across all study villages at the start of the study had an impact on transmission and further highlight the greater impact that can be achieved by combining bed nets with house-modification interventions, such as EaveTubes and household screening. Several studies have indicated that despite high levels of pyrethroid resistance, pyrethroid-treated bed nets can still offer some protection from infection [3, 16, 17]. Timing the endline survey to coincide with the same time of the malaria season as the baseline survey would have helped to see whether the bed nets had considerably reduced transmission in the control setting.

Infection prevalence was highest in children aged between 5 and 10 years old in both arms at endline. Together with children aged 10–15 years, these children were the least likely to have slept under a net—a finding that has been seen elsewhere in malaria endemic settings [18, 19]. However, despite the higher prevalence and lower use of bed nets, there was a reduction in prevalence in children living in intervention clusters for this age group compared to those in control villages, suggesting that being inside the house, even without a bed net, they were benefitting from the SET intervention in their village. An intervention which does not require user compliance, such as SET, has many advantages over those that may suffer from user fatigue or, as is often reported with bed nets, may not be used year round. The least impact of the intervention was seen in those aged between 25 and 50 years, despite high bed net use (77% reported using a net the previous night). It is possible that this age group benefits less from a house-based intervention due to behavioural patterns, e.g. through already having high bed net use which results in less of an add-on benefit of a house-based intervention, or due to remaining outside the house late at night when mosquito vectors are biting [20].

There were considerable discrepancies between the results of the RDTs and the results of the microscopy. One explanation for differences between the diagnostics could be that RDTs are still detecting circulating HRP-2 from previous infections; however, only a small proportion of participants reported receiving treatment for malaria in the previous 2 weeks, the majority of whom were either positive by RDT or both microscopy and RDT. Other potential reasons for discrepancies could be other circulating infections causing false positives in RDTs [21, 22] or, conversely, false negatives may be caused by parasites circulating with HRP-2 deletions, which would result in not being detected by RDT- HRP-2 deletions have been detected in parasites in neighbouring Ghana [23, 24].

One of the barriers to undertaking large scale trials, such as the Screening + EaveTubes trial in Côte d’Ivoire, is the limited number of funders who are willing or able to fund large cluster randomised control trials. Malaria case incidence can be an expensive and logistically challenging metric to collect, whereas prevalence is considerably cheaper to measure than incidence, due to the nature of the data collection (continuous monitoring of cohorts for incidence vs one off survey for prevalence). To illustrate efforts involved in active case detection for the incidence measure in this trial, the team undertook over 60,000 visits to approximately 2000 children over a 2-year period [11]. In this study, the proportion reduction in infection prevalence at the cluster level correlated strongly with malaria incidence, suggesting infection prevalence would have been a viable metric to evaluate SET in this setting. However, timing of the prevalence surveys needs to be carefully considered and the strength of correlation between prevalence and incidence seen in this setting may not be replicated in areas of lower transmission intensity. The strong correlation seen in this setting is perhaps surprising given the likely presence of super-infections (multiple infections at the same time) which would result in a non-linear relationship between the two metrics in high transmission settings, as has been predicted by mathematical models [25]. The relationship between incidence and prevalence is likely to differ with different age groups—incidence in this study was measured in children up to 10 years old, whilst prevalence was recorded across all age groups—however, when investigating the relationship between incidence and only children aged up to 10 years in the prevalence data, the relationship still appeared broadly linear (Additional file 2). Similarly, it could be hypothesised that interventions may have less impact on prevalence, compared to incidence, in areas where reinfection remains high—however, in this study, the impact on prevalence was of a similar magnitude to that seen on incidence. A more thorough examination of the empirical evidence of the relationship between prevalence, incidence, and indeed other metrics, such as entomological inoculation rate (EIR), at different transmission levels is needed to better understand which metric is most suitable for estimating impact of interventions.

## Conclusions

Epidemiological metrics are essential for a comprehensive evaluation of interventions and for those interventions to receive a recommendation from the WHO. In this high transmission setting, malaria case incidence and infection prevalence were similarly reduced in the SET intervention clusters, highlighting the usefulness of this intervention combination to reduce malaria transmission in this setting. Whilst the incidence measures collect interesting information regarding the differential impact over season and are likely the most useful metric in low transmission settings, infection prevalence may be a good option for more cheaply evaluating new products where transmission is moderate to high.

## Supplementary Information

**Additional file 1.** Map showing the location of the study villages in Bouaké, Côte d’Ivoire. The pie charts indicate the proportion of houses in each cluster that received the intervention.

**Additional file 2.** Graph showing the association between endline infection prevalence and malaria case incidence in children aged 6 months to 10 years.

## Data Availability

The datasets used and/or analysed during the current study are available from the corresponding author on reasonable request.

## Abbreviations

CI: Confidence interval
HRP-2: Histidine rich protein-2
ITN: Insecticide treated net
OR: Odds ratio
RDT: Rapid diagnostic test
SET: Screening and EaveTubes
