# Supplementary figures and images for: Housing modification for malaria control: impact of a “lethal house lure” intervention on malaria infection prevalence in a cluster randomised control trial in Côte d’Ivoire
Source: BMC Med. 2023 May 4;21:168. doi: 10.1186/s12916-023-02871-1 (PMC10161487; doi:10.1186/s12916-023-02871-1)

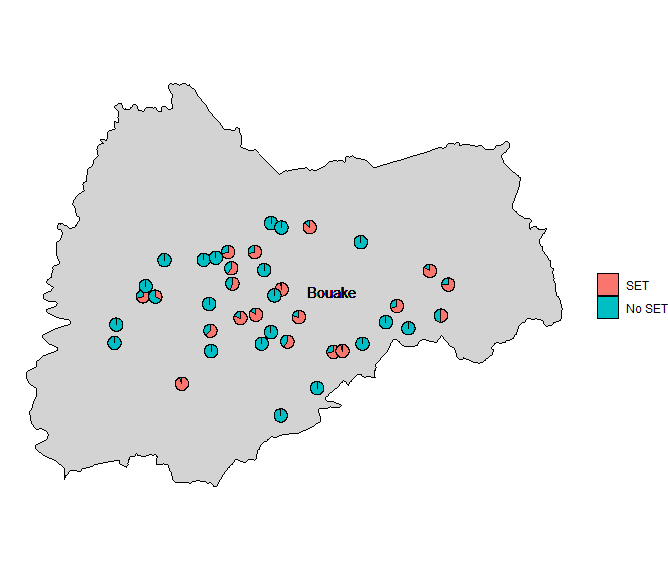

Supplement: Supplementary file 1 — Additional file 1. Map showing the location of the study villages in Bouaké, Côte d’Ivoire. The pie charts indicate the proportion of houses in each cluster that received the intervention. [file 12916_2023_2871_MOESM1_ESM.png]

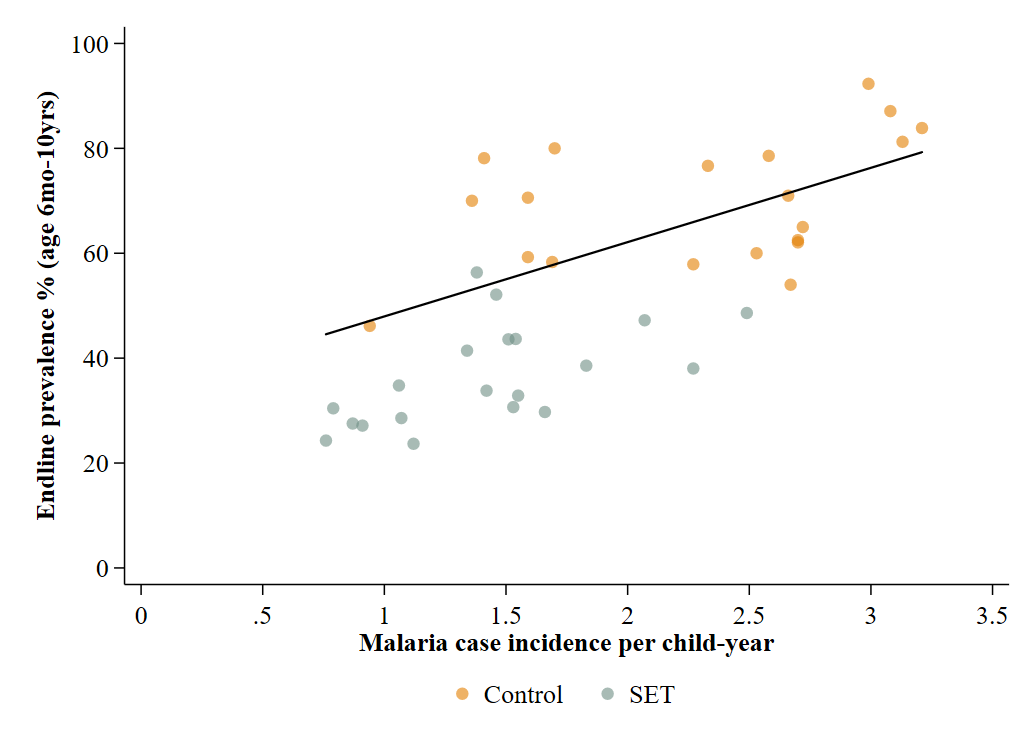

Supplement: Supplementary file 2 — Additional file 2. Graph showing the association between endline infection prevalence and malaria case incidence in children aged 6 months to 10 years. [file 12916_2023_2871_MOESM2_ESM.png]
